# Locking Plate Alone or in Combination with Cannulated Screws for Hoffa Fractures: A Retrospective Study

**DOI:** 10.1111/os.13201

**Published:** 2022-01-30

**Authors:** Zhen Li, Zhenyue Chen, Xiaotan Wang, Jingyin Li, Lizhong Jing, Zehui Li, Xuewei Cao

**Affiliations:** ^1^ The Second Clinical Medical College of Guangzhou University of Chinese Medicine Guangzhou China; ^2^ The First Clinical Medical College of Guangzhou University of Chinese Medicine Guangzhou China; ^3^ The First Clinical School, Shandong University of Traditional Chinese Medicine Jinan China; ^4^ Department of Orthopaedics the First Affiliated Hospital of Shandong University of Traditional Chinese Medicine Jinan China; ^5^ Department of Orthopaedic Surgery Guangdong Provincial Hospital of Traditional Chinese Medicine Guangzhou China

**Keywords:** Hoffa fractures, locking plate, cannulated screws, early rehabilitation

## Abstract

**Objective:**

To determine the efficacy of distal femur condyle locking plate (DFCLP) alone or in combination with cannulated screws for Hoffa fractures.

**Methods:**

In this study, between May 2014 and February 2019, 13 patients between 26 and 64 years with isolated Hoffa fractures were enrolled during the study period and retrospectively analyzed. All patients underwent open reduction and internal fixation by DFCLP alone or in combination with cannulated screws followed by early active rehabilitation postoperatively. The primary outcome was evaluated using range of movement (ROM), Knee Society Score (KSS), International Knee Documentation Committee (IKDC) scoring system, and the fracture healing time of the patients during the 24‐month follow‐up period. Postoperative complications were also used to assess the patients’ conditions.

**Results:**

A total of 13 patients completed the 24‐month follow‐up assessment and achieved bone re‐union at Hoffa fracture sites. The average follow‐up period was 24.5 months (ranging from 24 to 28 months). Six patients were treated by DFCLP in combination with cannulated screws and the remaining seven patients were treated by DFCLP alone. The mean ROM was determined as 119° (ranging from 100° to 130°). The mean KSS score was 87.9 (ranging from 80 to 92 points), with 11 patients evaluated as excellent, two as good, and zero bad cases. The mean IKDC score was 84.2 (ranging from 74.7 to 89.7 points), with 10 evaluated as excellent, three as good, and zero bad cases. The mean IKDC score was 83.3 for patients with medial Hoffa fractures and 84.4 for those with lateral Hoffa fractures. The average time to healing was 3.5 months (ranging from 3 to 4 months), and at month 3, the fracture healing was evident in seven patients (54%), and at month 4, fracture healing was seen in six patients (46%). It is worth mentioning that two patients suffered from knee joint stiffness and osteoarthritis during the 24 months follow‐up. Eleven patients (84.6%) achieved satisfactory knee joint function through early postoperative rehabilitation.

**Conclusion:**

In patients with Hoffa fractures, treatment with DFCLP alone or in combination with cannulated screws followed by early active rehabilitation resulted in great stability and satisfactory functional outcomes after 24 months. Our findings may provide surgeons with a new way to treat Hoffa fractures.

## Graphical abstract

(Left) A 60‐year‐old female patient suffered from Hoffa fracture due to a high‐velocity fall. CT of left knee showed a type Letenneur III medial Hoffa fracture (A, B). Radiographs showed that the patient underwent open reduction and internal fixation, and a DFCLP and four cannulated screws were placed at the medial distal femoral condyle (C, D). The patient could acquire nearly 0°‐130° range of motion after 24 months postoperatively with a good functional recovery (E, F). (Upper right) A 52‐year‐old male patient sustained a left Hoffa fracture in a motor vehicle accident. Lateral radiograph and CT of left knee showed a type Letenneur III lateral Hoffa fracture (A–C). Radiographs showed that the patient underwent open reduction and internal fixation, and a DFCLP alone was placed at the lateral distal femoral condyle (D, E). Twenty‐four months after the operation, nearly 0°–120° range of motion was obtained in the patient with satisfactory functional results (F, G). (Lower right) Schematic illustration of anatomic locking plate on distal femur lateral condyle (A) and medial condyle (B). Ten fix screw holes are evenly distributed on the condyle, which could better fix the fracture fragments. Eight of them are arranged in arc along the boundary and could fix the joint surface effectively.

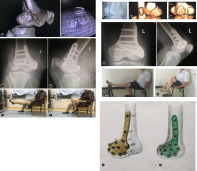

## Introduction

Hoffa fractures, also known as posterior coronal plane shear fractures of the femoral condyle, are rare injuries accounting for only around 8.7%–13% of distal femur fractures and most often occur in young adults^1^, ^2^. They were originally described by Friedrich Busch and then named in 1904 by Albert Hoffa^1^, ^2^, ^3^. It is an unstable intra‐articular fracture that is involved in 17% of supracondylar and intercondylar fractures^3^.

Hoffa fractures are most commonly associated with high‐energy trauma and typically occur when the knee is in a flexed position with excessive axial forces resulting in shearing of condyles, particularly in motor vehicle accidents and falls from a height^4^, ^5^. These fractures might be overlooked in standard anteroposterior and lateral radiographic examinations, particularly if the fracture is nondisplaced. It could be associated with further displacement if not identified soon. Therefore, computed tomographic (CT) scans are the recommended imaging modality for diagnosis, as they can offer a high degree of accuracy^3^. The purpose of the treatment of this kind of articular fracture is to achieve anatomical reduction. Nonoperative treatment is no longer recommended because of worse functional outcomes and increased rates of displacement^4^, whereas open reduction and internal fixation combined with early rehabilitation is the ideal treatment approach for better functional outcomes for Hoffa fractures^5^.

Nowadays, various surgical approaches and fixation methods have been established and widely practiced. Most surgeons prefer to use cannulated screws in the fixation of these fractures, while a number of studies are now examining locking buttress plates^1^, ^2^, ^3^, ^4^, ^12^, ^15^. However, no standard treatment methods exist for Hoffa fractures. Furthermore, the unstable nature of Hoffa fractures could result in fracture re‐displacement, internal fixation failure, and non‐union^3^, ^4^, ^5^, ^6^, increasing the patient's misery and economic burden. In our study, distal femur condyle locking plate (DFCLP) specially designed for Hoffa fractures, could achieve enough stability. To date, no literature on this type of fixation method of Hoffa fractures exists.

The purpose of this study is: (i) to evaluate the clinical performance of surgical treatments for Hoffa fractures; (ii) to assess whether there are better functional outcomes fixed by DFCLP alone or in combination with cannulated screws; and (iii) to discuss the superiority in application of early rehabilitation postoperatively.

## Methods and Materials

### Inclusion and Exclusion Criteria

Inclusion criteria were as follows: (i) patients aged between 26 and 64 years with isolated Hoffa fractures; (ii) patients who had undergone surgery fixed by DFCLP alone or in combination with cannulated screws followed by early active rehabilitation; (iii) patients who had completed records of clinical data.

Exclusion criteria were as follow: (i) patients who had undergone conservative treatments; (ii) patients who had suffered open or pathological fractures; (iii) patients who had any additional supra‐condylar or inter‐condylar fractures; (iv) patients who had severe neurovascular injury; and (v) patients who had undergone any previous surgery of the involved knee.

### Study Design

This retrospective study was approved by the ethics committee of Shandong University of Traditional Chinese Medicine Affiliated Hospital (no. 2019‐037‐01). Our work complies with the STROBE criteria. Using the orthopaedic trauma files in our institute, data were gathered for this retrospective study. Patients who underwent surgery with DFCLP fixation alone or in combination with cannulated screws for Hoffa fractures in our institution between May 2014 and February 2019 were reviewed. Informed consent was provided by all participants.

### Intervention

All patients underwent surgery with DFCLP fixation alone or in combination with cannulated screws followed by early active rehabilitation. The DFCLP and cannulated screws were provided by Jiangsu Jinlu Medical Company. Study subjects include 13 patients, the attendance on the follow‐up appointments were perfect, and no patients dropped out.

### Classification of Fracture

The fracture side, mechanisms of injuries, and follow‐up period were all recorded. All fractures were closed and categorized according to the Letenneur descriptions^7^. Of the 13 fractures, there were two Letenneur I fractures, four Letenneur II fractures, and seven Letenneur III fractures. The fractures included 10 lateral (77%) cases and three medial cases (23%). Misdiagnosis occurred in one (7.7%) patient who suffered from continuous pain and limited knee flexion, and 4 weeks elapsed since the initial injury to being diagnosed (Table 1).

**TABLE 1:** Demographic data of the patients

Case	Age/Gender	Injury mechanism	Fracture subtype	Surgical approach	Late diagnosis (weeks)	Follow‐up (months)
1	60/F	High‐velocity fall	III/MC	Medial	–	24
2	46/M	High‐velocity fall	IIb/LC	Lateral	4	24
3	61/M	Motor vehicle accident	I/LC	Lateral	–	26
4	37/M	Motor vehicle accident	IIb/MC	Medial	–	24
5	51/M	Heavy object smashing	IIa/LC	Lateral	–	24
6	52/M	Motor vehicle accident	III/LC	Lateral	–	28
7	64/F	High‐velocity fall	III/LC	Lateral	–	24
8	45/M	High‐velocity fall	IIa/LC	Lateral	–	24
9	46/M	Motor vehicle accident	III/LC	Lateral	–	24
10	26/F	Motor vehicle accident	I/LC	Lateral	–	24
11	63/F	High‐velocity fall	III/MC	Medial	–	24
12	54/M	High‐velocity fall	III/LC	Lateral	–	24
13	44/M	High‐velocity fall	III/LC	Lateral	–	24

Abbreviations: M, male; F, female; MC, medial condyle; LC, lateral condyle I, II, III: fracture classification of Letenneur I, II, III

### Surgical Techniques

#### Anesthesia and Position

All surgical procedures were performed by the same professional surgical team. Patients were under general or spinal anesthesia for the surgery, and tourniquets were used during the surgery to limit blood flow. Patients were placed supine on the operating table with the involved knee bent at 60° and a soft pad inserted under the popliteal fossa.

#### Approach

Open reduction through the modified medial or lateral approach was used to treat all fractures. The medial approach incision was made at the level of the femoral adductor tubercle, which was curved parallel to the tibial margin, and the incision was stopped at the lower margin of the tibial plateau. The sartorius anterior margin was identified first and separated along the space between itself and the vastus medialis. Then, the knee was flexed and pulled back the sartorius to expose the adductor magnus tendon and the adductor tubercle, and the adductor magnus tendon was pulled back last to expose the fracture. The lateral incision approach extends downwards along the lateral femoral axis across the lateral femoral condyle, turns to the tibial tubercle, enters along the anterior or posterior side of the iliotibial tract, cuts fascia in front of the lateral muscle septum, and pulls the lateral femoral muscle upward to expose the fracture and articular surface.

#### Internal Fixation

The hematoma was cleaned, and the articular surface reduced with forceps. The intact fragment of the Hoffa fracture was reduced and temporarily fixed by Kirschner wires in the anteroposterior or posteroanterior position. The direction of Kirschner wire was as far vertical to the fracture line as possible. Biplanar fluoroscopic imaging was used to confirm the reduction.

Afterward, DFCLP or cannulated screws were placed to fix fractures. The ideal screw directions would be perpendicular to the fracture line of the major coronal plane along the longest axis of the medial femoral condyle. There are many sizes of screws that can be used, depending on fracture fragment size, ranging from mini fragment implants (2.0 and 2.4 mm) to 3.5‐mm cortical screws, and 4‐ or 6.5‐mm cannulated screws. Meanwhile, DFCLP was placed on the side of the femoral condyle for preventing the fragment from gliding vertically as well as fixing it with angular stability. However, how the plate was placed should be evaluated accurately to prevent the screws from interfering with each other. Direct fluoroscopic visualization was then used to confirm the precision of the reduction and implant placement. The stability and motion range of the knee were checked, followed by the deflation of the tourniquet and closure of the wound over suction drains.

### Rehabilitation

One of the most crucial targets during the early stage of the postoperative rehabilitation was to minimize swelling and pain, thus elevation of the affected leg combined with non‐steroidal anti‐inflammatory drugs were recommended. When the pain and swelling subsided, the continuous passive motion (CPM) system was used for helping the knee joints to do extension and flexion exercises in all patients from postoperative day 3^2^. On postoperative day 7, the degree of knee flexion increased from 0° to 60°. Starting from the second week, the frequency of the exercise was increased to 2–3 times a day, to achieve a 10° to 15° advance in the range of knee flexion every day. By the end of postoperative week 3, patients were encouraged to reach a milestone of between 100° to 130°of flexion. In the fourth week, patients were allowed to perform a full range of motion (ROM) for 30 to 40 mins at a time, 2 to 3 times a day. During weeks 6 to 8 after surgery, patients began practicing walking without bearing weights using crutches, which is followed by partial weight‐bearing walks at around week 10. For patients who had been discharged, the weight born was gradually increased following the guidance of the clinician given through tele‐rehabilitation. Full weight‐bearing walks were allowed once signs of bone reunion were detected on radiographs from weeks 14 to 28 postoperatively.

### Clinical and Radiological Assessments

The ROM was used to evaluate the knee flexion and extension postoperatively. Subjective function scores, including the Knee Society Score (KSS) and the International Knee Documentation Committee (IKDC) score, were used to evaluate the improvement in knee function. Any stability, type of fixation, fracture healing time, and complications were noted. The details are presented in Table 2. The clinical assessments postoperatively were collected at last follow‐up. An independent assessor who was unaware of the types of surgical treatment and fracture carried out all evaluations. Detailed radiological assessment of knee, including X‐rays and CT scans, were conducted preoperatively and postoperatively. After being discharged, patient follow‐up was carried out every month for the first 3 months using X‐ray or CT scans, then every 3 months in the following 9 months, and finally at the 12th and 24th months.

**TABLE 2:** Functional results and complications of the patients

Case	Fixation	Knee ROM (°)	Score		Fracture healing time (months)	Stability	Complications
			KSS	IKDC			
1	Plate and screws	0–130	88	88.5	3	Stable	‐
2	Plate	0–120	90	86.2	3	Stable	‐
3	Plate	0–125	91	87.4	4	Stable	‐
4	Plate	0–110	86	75.9	4	Stable	‐
5	Plate	0–115	85	82.8	3	Stable	‐
6	Plate	0–120	84	81.6	3	Stable	Stiffness
7	Plate and screws	0–120	91	89.7	4	Stable	‐
8	Plate	0–125	90	85.1	3	Stable	‐
9	Plate and screws	0–100	80	74.7	4	Stable	Stiffness osteoarthritis
10	Plate	0–120	88	83.9	3	Stable	‐
11	Plate and screws	0–120	89	85.6	3	Stable	‐
12	Plate and screws	0–125	92	87.4	4	Stable	‐
13	Plate and screws	0–120	90	85.2	4	Stable	‐

Abbreviations: IKDC, International Knee Documentation Committee Subjective Knee Form Score; KSS, Knee Society Clinical Score; ROM, range of motion.

#### Range of Motion (ROM)

Normal knee ROM usually refers to how much the knee bends and straightens. The most accurate way to measure knee ROM is to use a goniometer. A goniometer is essentially a specially designed protractor that measures joint angles. Place the axis of the goniometer over the lateral femoral epicondyle. Line the stationary arm of the goniometer up with the greater trochanter along the outer thigh. Line the other arm of the goniometer up with the lateral malleolus of the ankle. Knee flexion and extension ROM is necessary for functional and sport‐specific activities^25^.

#### Knee Society Score (KSS)

Knee function was evaluated at last follow‐up using the KSS, which consists of knee score and function score, each with a maximum of 100 points. The knee score rates the knee joint based on pain, stability, and ROM, with deduction for flexion contracture, extension lag, and malalignment. The function score rates the patient's ability to walk and climb stairs, with deduction for aids^26^.

#### Knee Documentation Committee Subjective Knee Form (IKDC) Score

The International Knee Documentation Committee Subjective Knee Form (IKDC) was designed to assess patients with a variety of knee disorders including ligamentous and meniscal injuries as well as patellofemoral pain and osteoarthritis. The IKDC is a patient‐completed tool, which contains sections on knee symptoms (seven items), function (two items), and sports activities (two items). Scores range from 0 points (lowest level of function or highest level of symptoms) to 100 points (highest level of function and lowest level of symptoms)^25^.

#### Fracture Healing Time

Fracture healing is defined as (i) no tenderness or longitudinal percussion pain at the fracture site; (ii) no abnormal movement at the fracture site; and (iii) X‐ray showing blurred or absent fracture line with continuous callous passing through the fracture site. The normal healing time of Hoffa fracture is 3–4 months. The X‐ray and CT scans were used to assess the healing of the fractured site. This parameter was used to evaluate the presence of bone delayed union or nonunion.

#### Surgical Complications

Surgical complications were collected using information documented in the medical records. Surgical complications were defined based on definitions of surgical complications reported in the literature, including implant loosening, superficial and deep infections, knee stiffness, periprosthetic fractures, ligament injuries, hardware failure, and wound healing problems^27^.

ROM, KSS scores, and IKDC scores were often used to evaluate the clinical function of knee surgery, and to assess the direct effect of surgery from different angles. Even if affected by many factors, the healing time was the most critical step in the treatment process, followed by clinical function. Surgical complications were a parameter to evaluate the prognostic effect, which together with clinical function parameter and healing time form a complete evaluation system.

### Statistical Analysis

Demographic data of the patients and clinical scores are shown as mean. Percentages are also used to illustrate the proportions of the statistics. All statistical analyses were conducted using SPSS 23.0 for Windows (IBM, Armonk, NY, USA).

## Results

### General Results

Initially, data from patients who had suffered from intra‐articular distal femur fractures were identified through searching from a prospective orthopaedic database, out of which there were 19 cases (2.89%) with Hoffa fractures. After applying the inclusion criteria, a total of 13 patients who had suffered isolated Hoffa fractures were enrolled in the study. The patient sample comprised of nine males and four females with an average age of 49.9 years (range: 26–64 years) at the time of surgeries. The general patient demographic data are shown in Table 1.

Patients who participated in this study were all cooperative and the full clinical data were gathered. The bone reunion was observed in all patients on their Hoffa fractures sites, with the articular surface of femoral condyles reduced anatomically. The average follow‐up period was 24.5 months (ranging from 24 to 28 months). Six patients were treated with DFCLP in combination with cannulated screws and the remaining seven patients were treated with DFCLP alone. Functional results and complications of the patients are detailed in Table 2.

### Knee Functional Outcomes

#### Range of Motion (ROM)

The mean ROM was 119° (ranging from 100°to 130°). Ten patients (76.9%) had more than 120° of postoperative ROM. Only one patient (7.7%) had postoperative ROM less than 110°.

#### Knee Society Score (KSS)

The mean KSS score was 87.9 (ranging from 80 to 92 points) with 11 patients evaluated as excellent, two as good, and zero bad cases. The mean KSS was 87.7 for patients with medial Hoffa fractures and 88.1 for those with lateral Hoffa fractures.

#### Knee Documentation Committee Subjective Knee Form (IKDC) Score

The mean IKDC score was 84.2 (ranging from 74.7 to 89.7 points), with 10 evaluated as excellent, three as good, and no bad cases. The mean IKDC score was 83.3 for patients with medial Hoffa fractures and 84.4 for those with lateral Hoffa fractures.

#### Fracture Healing Time

The X‐ray image and CT scans were used to assess the healing of the fractured site. The results indicated that at month 3, the fracture healing was evident in seven patients (54%), and at month 4, fracture healing was seen in six patients (46%). In the end, all fractures had healed, and none of the patients developed delayed union or nonunion. Knee joint stability was achieved in all patients.

#### Surgical Complications

Two patients suffered from knee joint stiffness and osteoarthritis during the follow‐up period. Eleven patients (84.6%) in total regained satisfactory function of their knee joint and ability to walk with decent clinical results obtained through early postoperative rehabilitation. Additionally, no limb‐length discrepancy, non‐union, postoperative infection, bone resorption, or secondary displacement happened, and patients were all free from pain and able to walk without any aid.

#### Typical Cases

**Fig. 1:**
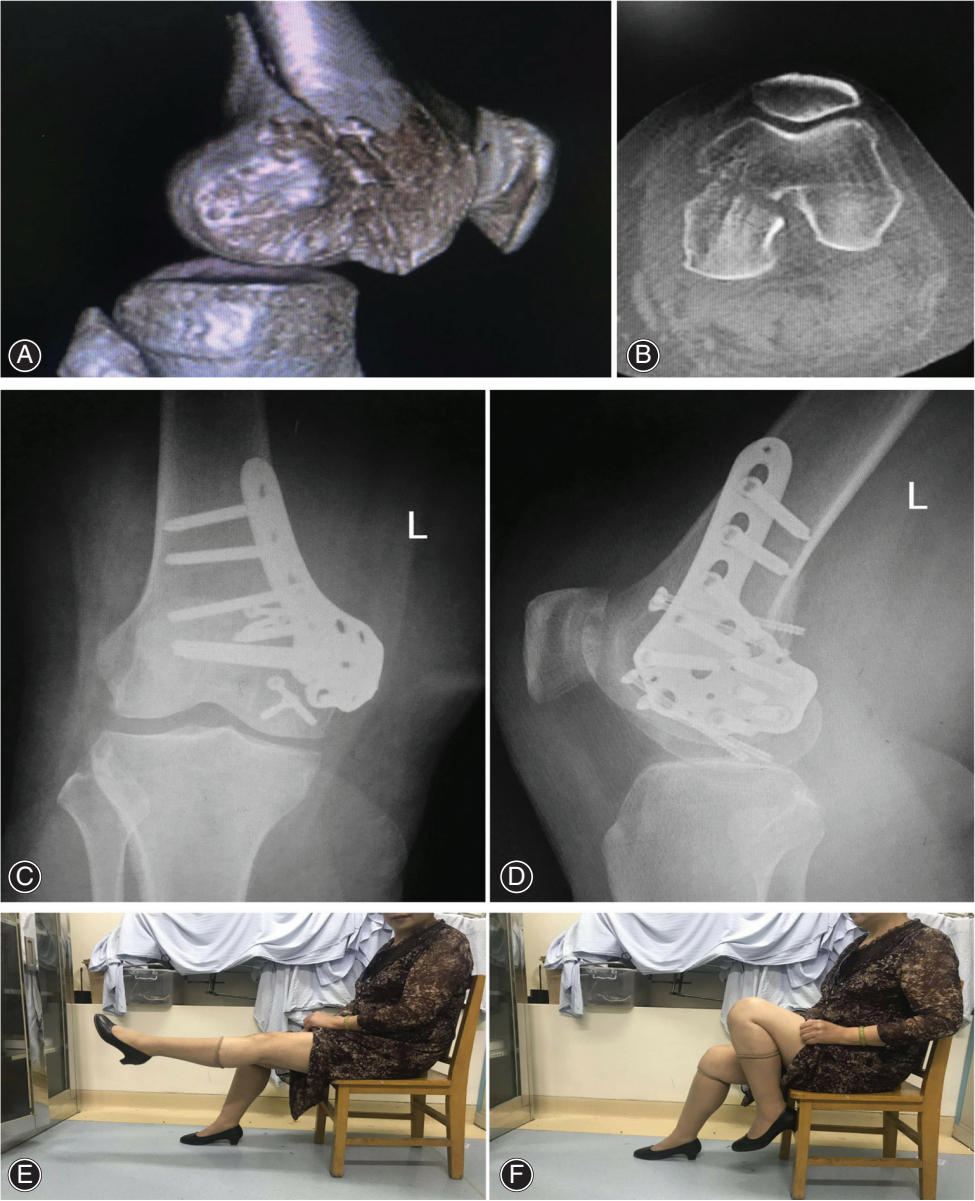
A 60‐year‐old female patient suffered from Hoffa fracture due to a high‐velocity fall. CT of left knee showed a type Letenneur III medial Hoffa fracture (A, B). Radiographs showed that the patient underwent open reduction and internal fixation, and a DFCLP and four cannulated screws were placed at the medial distal femoral condyle (C, D). The patient could acquire nearly 0°–130° range of motion after 24 months postoperatively with a good functional recovery (E, F)

**Fig. 2:**
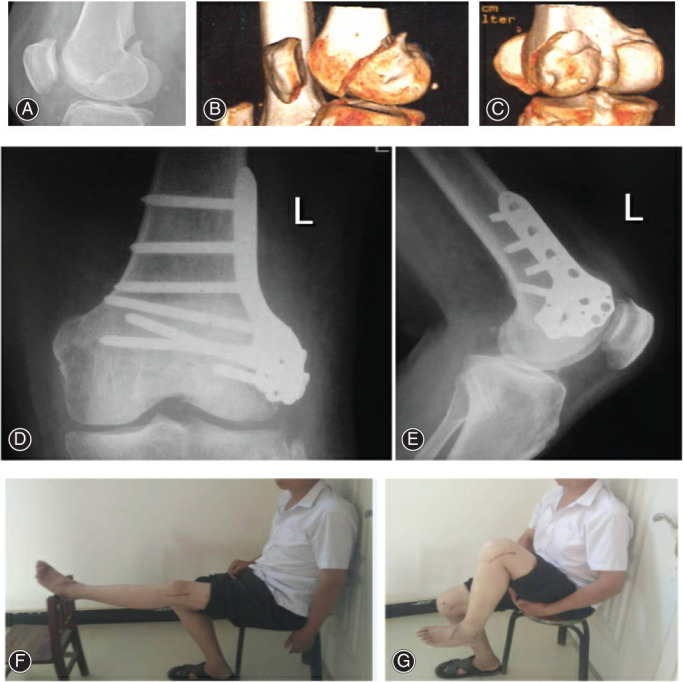
A 52‐year‐old male patient sustained a left Hoffa fracture in a motor vehicle accident. Lateral radiograph and CT of left knee showed a type Letenneur III lateral Hoffa fracture (A–C). Radiographs showed that the patient underwent open reduction and internal fixation, and a DFCLP alone was placed at the lateral distal femoral condyle (D, E). Twenty‐four months after the operation, nearly 0°–120° range of motion was obtained in the patient with satisfactory functional results (F, G)

In our study, the patient of case 1 (a 60‐year‐old female) got the left medial Hoffa fracture (type Letenneur III) from a high‐velocity fall. A DFCLP and four cannulated screws were placed at the medial distal femoral condyle. Twenty‐four months after the operation, nearly 0°‐130° ROM was obtained in the patient with a good functional recovery (Figure 1). Another patient (case 6, a 52‐year‐old male) suffered the left lateral Hoffa fracture (type Letenneur III) from a motor vehicle accident. A DFCLP alone was placed at the lateral distal femoral condyle. Twenty‐four months after the operation, nearly 0°‐120°ROM was obtained in the patient with satisfactory functional results (Figure 2).

## Discussion

### Main Findings

The main findings of the present study were that DFCLP alone or in combination with cannulated screws followed by early active rehabilitation were efficacious in providing stability and improving functional outcomes after 24 months in patients with Hoffa fractures, which is the special point of this study. The ROM, KSS scores, and IKDC scores in all patients were significantly improved. In addition, the radiographic assessments demonstrated the excellent healing time. Hoffa fracture healing was achieved in all patients with an average time to healing of 3.5 months. No severe complications occurred at follow‐up.

### Related Study

Previous research has demonstrated that Hoffa fractures involving medial condyles were even rarer than the lateral condyle^2^, ^5^, ^12^. This present study recruited 10 lateral and three medial Hoffa fractures, which may be thought to be the consequences of the particular injury mechanism involving the genu valgum in the knee joint as explained earlier^2^, ^5^, ^9^, ^10^, ^11^, ^12^. According to White *et al*.^6^, the knee was placed at 90° or more in a flexed position, hence the femoral condyle would be more prone to be subjected to direct anteroposterior force, thereby inducing Hoffa fractures. Furthermore, high‐energy trauma is the typical injury mechanism mostly seen in the setting of motor vehicle accidents and high‐velocity falls in our study. In addition, in the present study, one (7.7%) patient who presented with continuous pain and limited knee flexion was diagnosed with a Hoffa fracture 4 weeks after the initial injury. Thus, the accurate diagnosis of Hoffa fractures is challenging, and it is often overlooked on plain radiographs. Nork *et al*.^3^ highly recommended preoperative CT scans to detect any associated coronal fractures. Onay *et al*.^5^ also suggested the crucial need of CT scans to distinguish coronal fractures more accurately as these fractures can be easily neglected on plain radiographs. It is believed that CT scans are the best method for diagnosing and improving the diagnostic yield when there is clinical and radiological suspicion of Hoffa fractures.

Previous research has shown that there is no consensus on the most suitable surgical approach and fixation device so far^10^, ^11^, ^12^. In our study, the direct lateral approach was performed in 10 patients and the medial approach was performed in three patients. Six patients were treated with DFCLP in combination with cannulated screws, and the remaining seven patients were treated with DFCLP alone.

Nowadays, locking plate or screws have been recommended for Hoffa fractures^15^, ^16^, ^17^, ^18^. For screws, previous studies have shown that the anterior–posterior (AP) fixation is suitable for Letenneur I and III, which has larger fracture fragments^8^, ^9^, ^19^, ^20^, ^21^, ^22^, ^23^. The screws should be parallel to the slope of the medial and lateral femoral condyles, 25° to the medial condyle, and 10° to the lateral condyle^12^, ^13^, ^14^. Furthermore, it is generally accepted to have screw heads recessed beneath the articular surface. On the other hand, posterior–anterior (PA) fixation is suitable for type II with smaller fracture fragments^9^. However, it is often accompanied by a higher risk of damage to nerves and blood vessels. Onay *et al*.^5^ reported that screw fixation was seen to provide enough biomechanical stability until the fracture healed. In addition, Xu *et al*.^28^ proposed the triangular cross fixation with three screws for the treatment of Hoffa fractures, which proved to be as effective as the traditional fixation with two screws for parallel compression. However, in some cases, screw fixation alone is insufficient to resist the plane force, varus and valgus stress of the knee joint during flexion and extension, and it is often accompanied by a higher failure rate^8^, ^16^, ^17^, ^18^. Therefore, most studies recommended plate alone or in combination with cannulated screws as the ideal treatment modality. Gao *et al*.^2^ reported that the use of posterior anti‐sliding plate could enhance the fixation stability and allow early rehabilitation. Lu *et al*.^21^ found that fixation with compression screws and buttress plate may provide more adequate stability and better outcomes *vs* compression screws only. Agarwal *et al*.^24^ reported that lateral buttress plate along with cancellous screw fixation for Hoffa fracture provided stable fixation and a satisfactory final functional outcome.

### Functional Outcomes

In the present study, we used the lateral buttress plate, DFCLP, alone or in combination with cannulated screws to treat Hoffa fractures, which could provide sufficient stability of the fixation and permit early rehabilitation. A systematic plan of early active rehabilitation was carried out to achieve maximum restoration of the injured knee joint. For correcting knee flexion and extension deficits, the early rehabilitation protocol is indispensable. The advantages of early rehabilitation could reduce the rate of the postoperative complications, which include lowering the odds of acquiring Deep Vein Thrombosis (DVT), joint stiffness, and postoperative infection^2^. As reported by Gao *et al*.^2^, locking plate and screws for Hoffa fracture gave satisfactory functional results when coupled with aggressive rehabilitation. In our study, all the patients were compliant and received positive rehabilitation during the follow‐up period. In the end, 11 patients (84.6%) regained sufficient knee joint function and ability to walk with decent clinical results through this fixation method and early postsurgical rehabilitation. The postoperative functional outcomes of all patients have been greatly improved with a mean ROM of 119°, a mean KSS scores of 87.9, and a mean IKDC scores of 84.2. Hoffa fracture healing was achieved in all patients with an average time to healing of 3.5 months. Meanwhile, surgical complications were not discovered, except for two patients who suffered from knee joint stiffness and osteoarthritis during the 24‐month follow‐up. The functional outcomes we got are similar to previous research results^2^, ^5^, ^12^.

### DFCLP Characteristics

**Fig. 3:**
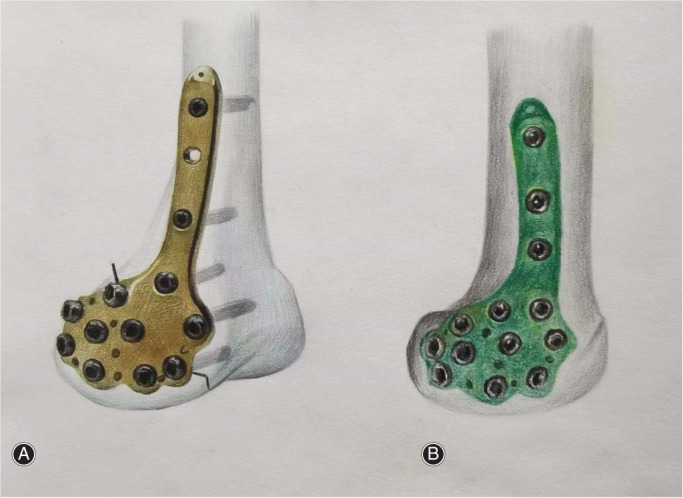
Schematic illustration of anatomic locking plate on distal femur lateral condyle (A) and medial condyle (B). Ten fix screw holes are evenly distributed on the condyle, which could better fix the fracture fragments. Eight of them are arranged in arc along the boundary and could fix the joint surface effectively

In addition, many advantages are reflected in DFCLP for the treatment of Hoffa fractures (Figure 3). First of all, it could better fit the surface of lateral condyle of distal femur due to its anatomic characteristics. Secondly, 10 fix screw holes are evenly distributed on the condyle, which could better fix the fracture fragments. Eight of them are arranged in arc along the boundary and could fix the joint surface effectively. Thirdly, multiplanar fixation via 3.5‐mm screws could improve the stress and stability. Moreover, there are six sutural pores in the distal plate, which can reinforce the suture of the joint capsule and small fragments, improving the stability. Finally, the proximal plate presents a slope inserting design, so the proximal screws could be fixed through minimally invasive approaches.

### Limitations

There were several limitations of our study. First, it was a retrospective study, which may weaken the strength of our conclusions. Second, the sample size was relatively small given the relative rarity of the pathological process that was studied. Third, the enrolled patients of the present study did not include Letenneur II type C fracture, which makes the study flawed. Fourth, our follow‐up period was a little short, only 24 months. Thus, further high‐quality research with a larger sample size is necessary to verify the current results. However, our study did have several strengths. To the best of our knowledge, this is the first study showing this type of fixation method of Hoffa fractures. Second, much importance is attached to the early rehabilitation of Hoffa fractures. Third, it contained a homogeneous group of patients from a single institution, all of whom were operated on by surgeons with similar operative indications and surgical techniques. Our findings may provide surgeons with a new way to treat Hoffa fractures.

### Conclusion

In conclusion, our results showed that DFCLP alone or in combination with cannulated screws followed by early active rehabilitation were efficacious in providing stability and improving functional outcomes after 24 months in patients with Hoffa fracture. Our findings may provide surgeons with a new way to treat Hoffa fractures.

## Data Availability

All data analyzed in this study has been provided in the manuscript and can be deposited publicly.
